# Multi-omics analysis of the gut microbiome and metabolites associated with the psychoneurological symptom cluster in children with cancer receiving chemotherapy

**DOI:** 10.1186/s12967-024-05066-1

**Published:** 2024-03-09

**Authors:** Jinbing Bai, Ronald Eldridge, Madelyn Houser, Melissa Martin, Christie Powell, Kathryn S. Sutton, Hye In Noh, Yuhua Wu, Thomas Olson, Konstantinos T. Konstantinidis, Deborah W. Bruner

**Affiliations:** 1 Nell Hodgson Woodruff School of Nursing, Emory University, 1520 Clifton Road NE, Atlanta, GA 30322 USA; 2 Winship Cancer Institute, Emory University, Atlanta, GA USA; 3 Aflac Cancer and Blood Disorders Center, Children’s Healthcare of Atlanta, Atlanta, GA USA; 4 School of Medicine, Emory University, Atlanta, GA USA; 5 School of Civil and Environmental Engineering, Georgia Tech, Atlanta, GA USA

**Keywords:** Children, Gut microbiome, Metabolome, Solid tumor, Chemotherapy, Multi-omics, Gut–brain axis

## Abstract

**Background:**

Children with cancer receiving chemotherapy commonly report a cluster of psychoneurological symptoms (PNS), including pain, fatigue, anxiety, depression, and cognitive dysfunction. The role of the gut microbiome and its functional metabolites in PNS is rarely studied among children with cancer. This study investigated the associations between the gut microbiome–metabolome pathways and PNS in children with cancer across chemotherapy as compared to healthy children.

**Methods:**

A case–control study was conducted. Cancer cases were recruited from Children’s Healthcare of Atlanta and healthy controls were recruited via flyers. Participants reported PNS using the Pediatric Patient-Reported Outcomes Measurement Information System. Data for cases were collected pre-cycle two chemotherapy (T_0_) and post-chemotherapy (T_1_), whereas data for healthy controls were collected once. Gut microbiome and its metabolites were measured using fecal specimens. Gut microbiome profiling was performed using 16S rRNA V4 sequencing, and metabolome was performed using an untargeted liquid chromatography–mass spectrometry approach. A multi-omics network integration program analyzed microbiome–metabolome pathways of PNS.

**Results:**

Cases (n = 21) and controls (n = 14) had mean ages of 13.2 and 13.1 years. For cases at T_0_, PNS were significantly associated with microbial genera (e.g., *Ruminococcus*, *Megasphaera*, and *Prevotella*), which were linked with carnitine shuttle (p = 0.0003), fatty acid metabolism (p = 0.001) and activation (p = 0.001), and tryptophan metabolism (p = 0.008). *Megasphaera*, clustered with aspartate and asparagine metabolism (p = 0.034), carnitine shuttle (p = 0.002), and tryptophan (p = 0.019), was associated with PNS for cases at T_1_. Gut bacteria with potential probiotic functions, along with fatty acid metabolism, tryptophan, and carnitine shuttle, were more clustered in cancer cases than the control network and this linkage with PNS needs further studies.

**Conclusions:**

Using multi-omics approaches, this study indicated specific microbiome–metabolome pathways linked with PNS in children with cancer across chemotherapy. Due to limitations such as antibiotic use in cancer cases, these findings need to be further confirmed in a larger cohort.

**Supplementary Information:**

The online version contains supplementary material available at 10.1186/s12967-024-05066-1.

## Background

Children with cancer receiving intensive chemotherapy frequently report cooccurring psychoneurological symptoms (PNS), including pain, fatigue, anxiety, depression, and cognitive dysfunction [1]. Collectively, these symptoms are defined as the PNS cluster, which can develop up to 6 months after treatment and even continue into survivorship [2]. Unfortunately, poor management and treatment of PNS can significantly reduce a child’s quality of life (QOL) and future psychosocial functioning [3, 4].

A symptom experience framework presented by Hockenberry and Hooke identified multiple antecedents that influence children’s experience of PNS across cancer treatment, including personal (e.g., sex and developmental stage), environmental (e.g., child’s hospitalization), and disease-related (e.g., type of cancer, length of treatment, treatment frequency, and chemotherapy drugs) factors [5]. Subsequent literature proposed that the PNS cluster may share common biological mechanisms [6], such as proinflammatory cytokines (e.g., IL-6 and TNF-α), Hypothalamic–Pituitary–Adrenal (HPA) axis, and monoamine neurotransmission system [7–9]. Nevertheless, the biological mechanisms of the PNS cluster are still largely unknown in cancer populations, particularly in pediatric oncology [10]. Recently, investigations of the microbiome–gut–brain (MGB) axis [11, 12] suggest that the gut microbiome (i.e., a collection of microorganisms and their genomes in the gastrointestinal tract) can signal the brain via functional metabolites and activation of other pathways (e.g., neurotransmitters), ultimately resulting in PNS for patients with cancer receiving chemotherapy [13, 14].

Chemotherapy has the potential to negatively interfere the MGB axis through a diverse set of pathways, including dysregulating the diversity and composition of bacteria in lumen, altering the gut microbiome-derived metabolites, and activating neuroimmune signaling [11, 12, 15]. As a commonly used treatment modality in children with cancer, chemotherapy can potentially lead to PNS via the MGB axis. Although limited, promising work has demonstrated enriched abundance of *Bacteroides* among adult patients with low PNS and enriched abundance of *Blautia* for those with high PNS [16]. Additionally, adult patients with head and neck cancer with high PNS had higher abundance of gut microbial Bacteroidota, *Ruminiclostridium*, and *Tyzzerella* compared to those with low PNS, while patients with low PNS had higher abundance of *Lactococcus* and *Phascolarctobacterium* compared to those with high PNS [13]. However, the role of the gut microbiome in PNS for children with cancer (CWC) has yet to be elucidated [17].

Microbiome-derived metabolites represent the functional role of the gut microbiome, as they are the drivers of gut–brain communication and carry out signals of a disturbed gut microbiome [18]. Communications between the gut and the brain occur following a network of pathways involving key microbial metabolites, such as short-chain fatty acids (SCFAs) [19] and tryptophan for kynurenine pathway metabolism [18]. SCFAs are part of a group of key microbial metabolome pathways associated with psychological functioning [20]. Alterations in the SCFA metabolism can result in disturbances to the central nervous system [21], although the effects of SCFAs on PNS have primarily been studied in animal models [20]. Additionally, tryptophan, an essential amino acid, is another key metabolite in the MGB axis, with dual emphasis on the regulation of serotonin and melatonin synthesis, and the control of kynurenine pathway [18, 22]. Tryptophan must be obtained from dietary or microbial sources [18] and can be synthesized from chorismate by bacterial phyla Pseudomonadota, Actinomycetota, and Bacillota [23].

In humans, untargeted metabolomics analysis showed that increased pain was associated with decreased tryptophan, and increased fatigue was associated with decreased arachidonic acid [24] in women with breast cancer receiving chemotherapy. Targeted metabolomics analysis further indicated moderate-to-strong correlations between changes in pain and tryptophan, as well as between changes in depressive symptoms and serotonin levels [24, 25]. Decreased tryptophan, increased kynurenine, and subsequent altered tryptophan/kynurenine ratio were associated with a higher level of PNS among cancer survivors [25]. Among children with cancer receiving chemotherapy, fatty acids pathways were associated with pain, and both tryptophan and carnitine shuttle pathways were associated with the PNS cluster [14].

A growing body of preclinical studies support the impact of the gut microbiome and microbial metabolites on the gut–brain communications via neuronal, immunological, and endocrinological pathways [26]. However, research on this mechanistic pathway in the context of chemotherapy-related PNS is still very limited. Furthermore, current work primarily adopts single-omics approaches (e.g., microbiome analysis or metabolomics analysis independently) in human health and disease. On the other hand, multi-omics approaches provide an opportunity to examine multiple layers of molecules (e.g., microbiome and metabolites) [27] to interpret health outcomes. Thus, there is paucity of research regarding how the interrelationship between the gut microbiome and their metabolites can influence PNS among patients with cancer receiving chemotherapy. Considering the severe PNS burden among children with chemotherapy and the unknown biological mechanisms of PNS, uncovering the multi-omics biological pathways within the MGB axis will pave a way for precision medicine (e.g., diet and probiotic interventions) to manage and treatment-related psychoneurological toxicities among CWC.

The purpose of this study was to investigate the associations between the gut microbiome–metabolome pathways and PNS among CWC receiving chemotherapy (pre-cycle two chemotherapy [T_0_] and post-chemotherapy within 4 weeks [T_1_]) compared to a group of healthy children (HC). An integrative multi-omics approach (i.e., metabolomics coupled to amplicon microbiome data) was adopted to examine the interrelationship of PNS-associated microbial taxa and their functional metabolites in CWC across chemotherapy. This study adopted a multi-omics network integration program xMWAS [28] to analyze associations of microbiome–metabolome pathways with PNS.

## Material and methods

### Design and setting

This study adopted a case–control study design. After informed consent (with child assent) was obtained from parents, children aged 7–18 years with solid tumors were recruited from the AFLAC Cancer and Blood Disorder Center at Children’s Healthcare of Atlanta in Atlanta, Georgia. Age-, sex-, race-, and body mass index (BMI)-matched HC were recruited via flyers, online e-news blast, and ResearchMatch™ [29] in the Greater Atlanta Area. Approval was obtained from the Institutional Review Board at Emory University (IRB No. 00102775).

### Participants

This study included two groups of children: CWC (n = 21) and HC (n = 14). Eligible CWC were: (1) 7–18 years old, (2) diagnosed with solid tumors (e.g., sarcomas, excluding brain tumors), (3) those who received at least one cycle of chemotherapy, (4) receiving treatment at the Aflac Cancer and Blood Disorder Center at Children’s Healthcare of Atlanta, and (5) agreed to participate in the study. Age-, sex-, race-, and BMI-matched HC were included if they were: (1) 7–18 years old, (2) not on antibiotics within the past 4 weeks, and (3) not involved in interventions (e.g., dietary program) that may influence the gut microbiome and metabolome. CWC with stem cell transplant, or relapses, or brain tumors, or whole abdominal radiotherapy within the past 4 weeks were excluded. For both CWC and HC, those with cognitive impairment (determined by treating physicians and neuropsychologists with objective cognition testing) or chronic diseases (e.g., inflammatory bowel diseases) that affect the gut microbiome and metabolome were excluded.

### Measures

#### Gut microbiome

Fecal specimens were collected to analyze the gut microbiome. Following the Human Microbiome Project protocol [30], children were instructed to collect fecal samples using an at-home collection kit that has been tested in our project with > 80% compliance [31]. Parents received instructions to assist their child to collect fecal samples. The provided spoon was used to transfer an aliquot of fecal sample into the collection tube (Fisher Scientific LLC., Pittsburgh, PA). Subsequently, the tubes were capped, placed into a biohazard bag, and then packed into a padded, labeled freezer bag with an ice pack. Samples were immediately placed into a freezer until shipped via FedEx. The FedEx shipment took approximately 24 h (range 16–24 h). Once received at the Emory Nursing Biobehavioral Laboratory, fecal samples were stored at − 80 °C until DNA extraction and assaying.

#### Metabolites

Metabolomic profiling of fecal metabolites in the gut was conducted following a well-validated protocol at Emory Lipidomics & Metabolomics Core [32], which identifies localized metabolic processes in the large intestine, colon, and rectum [33, 34]. An average of 100 mg (range from 95 to 105 mg) fecal specimen was aliquoted for each sample for untargeted metabolomics analysis. The untargeted metabolomics approach was utilized to acquire data for species, annotating metabolites, and reviewing both known and unknown metabolic changes. An advantage to untargeted data is its hypothesis-generating nature, which provides a foundation for further analysis using targeted approaches [35].

#### PNS

Children reported their PNS (e.g., pain, fatigue, anxiety, depressive symptoms, and cognitive dysfunction) using the Pediatric Patient Reported Outcomes Measurement Information System (PROMIS) [36, 37]. All the PNS reported by PROMIS scales aligned with clinical anchors (i.e., low blood counts) in children with cancer [38] and anchor-based methods using expert or patient judgment suggested a minimally important difference of 3 points on the PROMIS T-score scale for children [39]. The various PROMIS scales utilized in this study were scored using a T-score with a reference mean of 50 (standard deviation [SD] = 10) by the Health Measures Scoring Center. Previous reliability testing of the PROMIS short form (PROMIS-SF) system in adolescents reported Cronbach’s α coefficients ranging between 0.88 and 0.96 for initial surveys and exceeding 0.91 for subsequent visits. Furthermore, Cronbach’s α coefficients for pooled PROMIS-SF data across all visits ranged from 0.91 to 0.97 [40].

##### Pain

The one-item PROMIS Pain Intensity Scale was used to evaluate the child’s pain within the previous 7 days, with scores ranging from “No pain at all” (0) to “Worst pain” (10). This scale has demonstrated great construct validity and good feasibility for use in children aged 7–18 years with cancer [38]. The 8-item PROMIS Pain Interference Scale-SF was used to assess the influence of pain on the child’s social, cognitive, emotional, physical, and recreational activities over the past 7 days. A higher total score of pain interference indicates more pain impact on the child’s life.

##### Fatigue

The 10-item PROMIS Fatigue Scale-SF was used to assess the child’s fatigue within the previous 7 days, with scores ranging from “Not at all” (0) to “Always” (4). This scale has demonstrated good construct validity for use in children ages 7–18 years treated for cancer [38].

##### Anxiety and depressive symptoms

The 8-item PROMIS Anxiety Scale-SF was used to assess the child’s fear, anxiety, and somatic symptoms within the previous 7 days. The 8-item PROMIS Depressive Symptoms Scale-SF assessed the child’s depressive symptoms within the previous 7 days. Scores for each item on both scales ranged from “Never” (0) to “Almost always” (4). Both scales have demonstrated good construct validity for use in children aged 7–18 years treated for cancer [38].

##### Cognitive function

The 7-item PROMIS Cognitive Function Scale-SF assessed perceived difficulties in cognitive abilities. Scores were on a 5-point scale, with a higher total score indicating higher cognitive dysfunction. This scale has demonstrated excellent internal consistency and item-scale correlations in children [41].

#### Demographic and clinical variables

Children’s demographics (e.g., age, sex, race/ethnicity, and BMI percentile) and health history (e.g., use of antibiotics and disease history) were reported by their parents during the clinical visit. Cancer and treatment-related variables (e.g., type of cancer, cancer stage, and cycle of chemotherapy) were either reported by parents or extracted from the electronic medical records.

### Collection procedure

All the data for CWC were collected pre-cycle 2 chemotherapy (T_0_) and post-chemotherapy (T_1_, with an average 2 weeks post-chemotherapy [range 1–4 weeks]). Children confront various stressors from tumor diagnosis, treatment plans, and painful procedures during the first cycle of chemotherapy. To reduce psychological burden for the family, pre-cycle two chemotherapy period was selected for consent and data collection, with a mean of 3.7 weeks (range 2–8) from the first cycle chemotherapy in our participants. An average of 6 months between T_0_ and T_1_ were reported in this study. Only one timepoint of data was collected for HC. Children with solid tumors receiving chemotherapy were recruited during their routine outpatient clinic visits. Clinical collaborators from Children’s Healthcare of Atlanta identified eligible patients while a member from our research team consented parents (or children) and assented age-eligible patients. All PROMIS questionnaires were distributed for children to complete, and parents were provided pictorial instruction on at-home fecal specimen collection. The electronic medical records of CWC were used to collect demographic, clinical, and health-related variables. For HC, all procedures were identical, excluding the use of electronic medical records.

### DNA extraction

Based on the Human Microbiome Project protocol, microbial DNA was extracted from fecal samples using the PowerSoil isolation kit (Mo Bio Laboratories, Carlsbad, CA, USA) at the Environmental Microbial Genomics Laboratory, Georgia Institute of Technology. 16S rRNA amplicon libraries were prepared for the 16S rRNA V4 gene region [42]. These 16S rRNA amplicons were generated using KAPA HiFi HotStart ReadyMix (KAPA Biosystems, KK2600) and primers specific to 16S V4 region of bacteria and indices were attached using the Nextera XT Index kit (Illumina, FC-131-1001). Clean-up was performed on the indexed libraries using AMPure XP beads. The 16S libraries were pooled in equal amounts based on fluorescence quantification. Each run included a control template to test for polymerase chain reaction (PCR) accuracy and possible contamination. Final library pools were quantitated via qPCR (Kapa Biosystems, catalog KK4824). The pooled library was sequenced on the Illumina miSeq system using miSeq v3 600 cycle chemistry (Illumina, catalog MS-102-3003) at a loading density of 8 pM with 20% PhiX at PE300 reads. The microbial sequencing produced paired-end sequences.

### High-resolution untargeted metabolomics (HRM)

An HRM protocol established at the Emory Lipidomics & Metabolomics Core was adopted for liquid chromatography–mass spectrometry (LC–MS) analysis. Metabolic features were extracted from fecal samples using a 1:1 mixture of Acetonitrile: Methanol. 200 µL 1:1 Acetonitrile: Methanol was added to 50 μL samples, which was vortexed for 3 s, incubated on ice for 30 min, and then centrifuged at 20,000×*g* for 10 min to pellet precipitated protein. The supernatant was then transferred to an amber autosampler vial for LC–MS analysis. For quality control, a pooled quality control sample was created by combining 5 µL of each sample extract into a separate vial. This sample was run in triplicate at the beginning, the end, and intermittently over the course of analysis. Next, an untargeted HRM approach was performed using an ID-X™ Tribrid™ mass spectrometer coupled to a Vanquish Ultra-High-Performance Liquid Chromatography (UHPLC, Thermo Fisher Scientific Inc., San Jose, CA). Metabolic features from the fecal extracts were resolved on a SeQuant ZIC-HILIC™ 3.5 μm, 100A 150 × 2.1 mm column. For chromatography, water was used as Solvent A and Acetonitrile as Solvent B, both of which contained 0.1% Formic Acid. 1 μL extract was injected into the LC–MS system for analysis. A full scan MS1 spectrum for each sample was obtained at resolution of 120,000 and mass-to-charge ratio (m/z) range 67–1000. The mass spectrometer was operated in both positive and negative ionization modes. Uniquely detected ions consisted of accurate mass m/z, retention time and ion abundance, referred to as m/z features. Data were processed using Thermo Compound Discoverer software, which scans our metabolic data against internal and external databases. Raw data was uploaded into the software with m/z values and retention times aligned. Signal intensities are normalized by the pooled quality control sample and corrected to compensate for any variation of signal for batch correction.

### Statistical analysis

T-scores of the Pediatric PROMIS scales were calculated for PNS (excluding pain intensity). For their respective PROMIS questionnaires, T-scores ≥ 50 indicated significant fatigue, anxiety, and depressive symptoms, while a T-score ≤ 45 indicated significant cognitive dysfunction [43]. Independent sample t-test was used to compare the PNS between CWC and HC; paired sample t-test was applied to compare the PNS between T_0_ and T_1_ for CWC.

QIIME 2 default parameters were used to analyze the composition of the gut microbiome [44]. 16S rRNA sequence quality was filtered with dada2 to infer ASVs. Using the Silva132 database with a 97% identity threshold, a Naive Bayes classifier was trained to assign our ASVs to taxonomy at the phylum and genus levels to integrate into analysis. Silva database was selected due to its checked quality and regular updates of aligned 16S subunit rRNA sequences for bacteria. Alpha diversity (within-sample diversity, i.e., Shannon, observed OTUs, Pielou_e, and Faith_PD) and beta diversity (between-sample diversity, i.e., Jaccard and unweighted UniFrac distance) parameters reported associations of the gut microbiome with PNS. Meanwhile, filtering of metabolic data was performed to remove m/z features with median coefficient of variation (CV) within technical replicates ≥ 75%. Only samples with Pearson correlation within technical replicates ≥ 0.7 were selected for downstream analysis. Metabolite intensities were log_2_-transformed, and quantile normalized. Metabolites associated with PNS were annotated by matching m/z and retention time to currently confirmed metabolites via standardized laboratory references or matching computationally using xmsAnnotator [45]. xmsAnnotator uses metabolic pathway associations, intensity profiles, retention time, mass defect, and adduct patterns to match m/z features to publicly available metabolic databases [45].

A multi-omics network integration program, xMWAS [28], was used to integrate, illustrate, and analyze the microbiome–metabolome multi-omics pathways associated with PNS. xMWAS estimates correlations between numeric features from multiple sources (e.g., microbiome and metabolites relative intensity, and PNS scores) and plots a network graph with nodes (features) and lines (correlations). xMWAS provides two levels of interpretation for the network. First, cluster analysis algorithms are used to identify highly correlated groups of nodes; these groups may represent correlated biological phenomena that can be functionally annotated via pathway analysis programs. Second, centrality algorithms used in network analysis (i.e., eigenvector centrality [46, 47]) are used to identify and score the most influential nodes of the network according to their position and number of connections. Higher centrality scores indicate more connections to other nodes which themselves have many connections in the network. The specifics of the xMWAS program are as follows: the program performs stratified multi-omics integration, meaning it constructs and analyzes network graphs stratified by user-specified groups (e.g., CWC T_0_ and T_1_ and HC). Pairwise data integration was performed using Partial Least Squares (PLS) regression allowing the xMWAS program to identify the optimal number of PLS components through cross-validation. The igraph R package generated the integrative network, while multilevel community detection clustered the network by optimizing cluster modularity, a common community identification algorithm. An eigenvector centrality score between 0 and 1 was calculated for each node in each network graph. The relative influence of specific microbe, metabolite, and symptom nodes between CWC and HC and between CWC T_0_ and T_1_ was calculated as the change in centrality scores. As xMWAS requires a researcher-specified Pearson correlation cutoff to model network links, we used > |0.50| with a p < 0.05. Subsequently, the specific taxa that corresponded to each microbial metabolite cluster were annotated. In this study, the xMWAS approach only used high abundance microbes, meaning an average abundance > 0.0001 and detected in > 50% of the samples.

Metabolic pathway enrichment analysis was performed on the metabolites in each microbiome–metabolite community to describe dysregulation of microbiome–metabolome pathways specific for each PNS. This was conducted using Mummichog 2.0 default parameters [48]. Mummichog constructs metabolic pathways by mapping the reference list to the KEGG database and searches for enrichment from the user-specified list. Additionally, Mummichog calculates a Fisher’s exact p-value for each metabolic pathway via permutation testing using repeated random sampling from the referenced list. The Benjamini–Hochberg FDR method [49] was selected for multiple testing correction based on a *q* value of 0.20. However, the FDR correction protects against type I error but it may exclude true positive microbes and metabolites (type II error) [50]. Therefore, multi-omics pathways associated with PNS were determined by a raw p value of 0.05 [32]. These analyses were implemented by R 4.1.0.

## Results

### Participants’ demographic and clinical characteristics

Thirty-five children were included in the analytic sample, comprising of 21 CWC and 14 HC (Table 1). The CWC group had a mean age of 13.2 years, with 81% having a prior surgical procedure and more than 75% diagnosed with sarcomas. CWC received combined chemotherapy drugs following standard protocols, primarily including Doxorubicin (n = 12, 57.1%), Ifosfamide (n = 9, 42.9%), Cisplatin (n = 9, 42.9%), Etoposide (n = 8, 38.1%), Methotrexate (n = 7, 33.3%), Vincristine (n = 7, 33.3%), and Cyclophosphamide (n = 7, 33.3%). The primary treatment protocols were AEWS0031 (n = 7) and AOST0331 (n = 7). The HC group had a mean age of 13.1 years.

**Table 1:** Participants’ demographic and clinical characteristics

Variables	Children with cancer		Healthy controls (n = 14)	p-value*
	T0 (n = 21)	T_1_ (n = 12)^c^		
Age in years, mean (SD)^a^	13.24 (3.85)	13.75 (3.22)	13.07 (3.97)	0.90
Age categories, n (%)^b^				0.68
7–11 years	8 (38.1)	4 (33.3)	5 (35.7)	
12–17 years	12 (57.1)	8 (66.7)	7 (50.0)	
≥ 18 years	1 (4.8)		2 (14.3)	
Age at diagnosis, mean (SD)	13 (3.69)		NA	
Sex, n (%)^b^				0.09
Male	14 (66.7)	9 (75.0)	5 (35.7)	
Female	7 (33.3)	3 (25.0)	9 (64.3)	
Race, n (%)^b^				0.96
White	14 (66.7)	8 (66.7)	12 (85.7)	
Black	3 (14.3)	1 (8.3)	1 (7.1)	
Other	4 (19.0)	3 (25.0)	1 (7.2)	
Ethnicity, n (%)^b^				0.80
Hispanic	3 (14.3)	2 (16.7)	3 (21.4)	
Non-Hispanic	17 (81.0)	10 (83.3)	11 (78.6)	
Other	1 (4.7)	0	0	
Health insurance, n (%)^b^			NA	
Public	11 (52.4)	7 (58.3)		
Private	10 (47.6)	5 (41.7)		
Cancer type, n (%)^b^			NA	
Sarcomas	16 (76.2)	9 (75.0)		
Other	5 (23.8)	3 (25.0)		
Surgery, n (%)^b^			NA	
Yes	17 (81)	9 (75.0)		
No	4 (19.0)	3 (25.0)		
Antibiotic use, n (%)^b^				< 0.001
Yes	18 (85.7)	11 (91.7)	0 (0)	
No	3 (14.3)	1 (8.3)	14 (100)	
Preterm birth, n (%)^b^				0.99
Yes	1 (4.8)	1 (8.3)	1 (7.1)	
No	20 (95.2)	11 (91.7)	13 (92.9)	
Obesity status, n (%)^b^				0.99
Yes	1 (4.8)	1 (8.3)	0	
No	20 (95.2)	11 (91.7)	14 (100)	
Diabetes, n (%)^b^				0.99
Yes	1 (4.8)	1 (8.3)	0	
No	20 (95.2)	11 (91.7)	14 (100)	
Asthma, n (%)^b^				0.64
Yes	3 (14.3)	3 (25.0)	1 (7.1)	
No	18 (85.7)	9 (75.0)	13 (92.9)	
Diary intake intolerance, n (%)^b^				0.99
Yes	2 (9.5)	1 (8.3)	1 (7.1)	
No	19 (90.5)	11 (91.7)	13 (92.9)	
Lactose intolerance, n (%)^b^				0.40
Yes	0	0	1 (7.1)	
No	21 (100)	12 (100)	13 (92.9)	
Probiotics use, n (%)^b^				0.26
Yes	3 (14.3)	1 (8.3)	0	
No	18 (85.7)	11 (91.7)	14 (100)	

T_0_: pre-cycle two chemotherapy; T_1_: post chemotherapy (completion of all chemotherapy); NA: not applicable; SD: standard deviation

*p-value refers to healthy controls vs. pre-cycle 2 chemotherapy for cancer cases

^a^Independent samples t-test (for continuous variables)

^b^Pearson *Chi-Square* or Fisher’s Exact test (for categorical variables) were used to compare demographics and clinical variables between children with cancer and healthy controls

^c^12 children with cancer completed the gut microbiome data were included

No significant differences were found between the two groups in age (df = 33, p = 0.90), sex (df = 1, p = 0.09), race (df = 2, p = 0.96), ethnicity (df = 2, p = 0.80), and obesity status (df = 1, p = 0.99). Additionally, they had no significant difference in other health-related variables (e.g., asthma, diabetes, lactose intolerance, and use of probiotics). However, CWC received more antibiotics (due to cancer treatment) compared to HC (df = 1, p < 0.001).

### Comparison of psychoneurological symptoms by study group

**Fig. 1:**
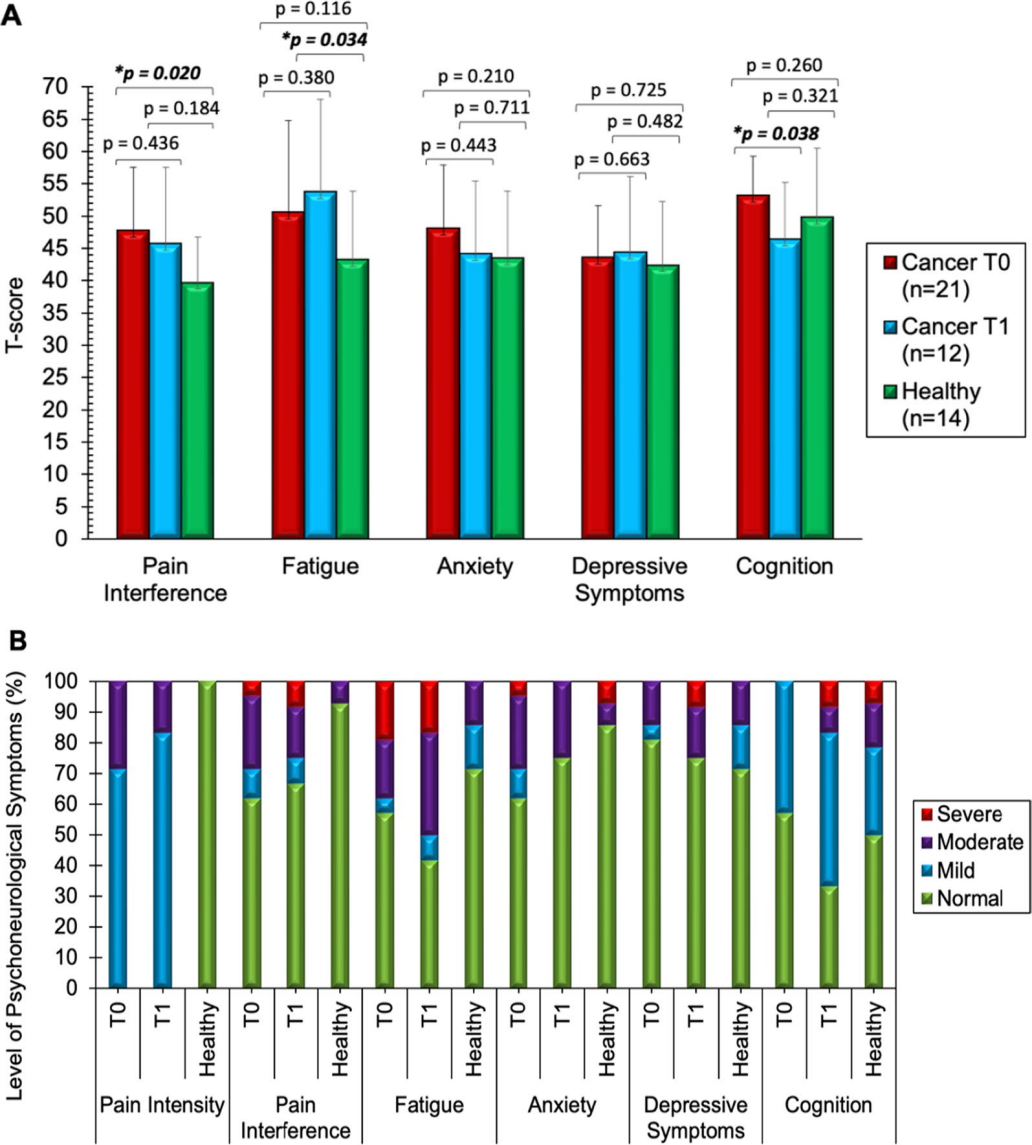
Comparison of psychoneurological symptoms between children with cancer and healthy controls. **A** Demonstrates T-scores of psychoneurological symptoms by mean ± standard deviation. **B** Demonstrates levels of psychoneurological symptoms by percentile (%) across cancer timepoint (T_0_ and T_1_) vs. healthy controls

Figure 1 describes the T-scores for individual PNS (pain interference, fatigue, anxiety, depressive symptoms, and cognitive dysfunction, Fig. 1A) and severity levels of PNS based on cutoff points of T-scores (Fig. 1B). CWC at T_0_ reported greater symptom burden as compared to HC, but statistical significance was only found for pain interference (mean [SD] = 47.77 [9.75] vs. 39.69 [7.06], df = 33, 95% confidence interval [CI] [1.36, 14.81], p = 0.020). Compared to CWC at T_0_, CWC at T_1_ showed lower pain (44.95 [12.10] vs. 47.77 [9.75], df = 11, 95% CI [− 4.43, 10.08], p = 0.436), lower anxiety (45.06 [11.32] vs. 48.05 [9.82], df = 11, 95% CI [− 4.78, 10.75], p = 0.443), higher depression (45.15 [12.02] vs. 43.57 [8.06], df = 11, 95% CI [− 8.83, 5.68], p = 0.663), higher fatigue (54.97 [14.38] vs. 50.61 [14.16], df = 11, 95% CI [− 5.57, 14.30], p = 0.380), although not statistically significant, but significantly worse cognitive dysfunction (46.40 [9.16] vs. 53.15 [6.14], df = 11, 95% CI [0.41, 13.10], p = 0.038). Fatigue among CWC at T_1_ was found significantly greater than in HC (54.97 [14.38] vs. 43.27 [10.59], df = 24, 95% CI [0.95, 22.46], p = 0.034). Figure 1B demonstrates similar trends in changes of PNS levels, showing CWC at T_1_ with more moderate fatigue and moderate/severe cognitive dysfunction than CWC at T_0_.

### Description of the gut microbiome and fecal metabolome

No significant difference was found for the total number of raw sequences across study groups (H = 3.97, df = 2, p = 0.137). Following the dada2 process, 1,572 features (or amplicon sequence variants [ASVs]) were identified, with a total frequency of 4,218,986 features. Frequencies per ASV ranged from 2 to 411,771, with a median frequency of 90, while ASV frequencies per sample ranged from 19,743 to 282,019, with a median frequency of 81,406. Rarefaction was not conducted because all study groups were plateaued around 4000 reads per sample in alpha-rarefaction curves. Using the trained classifiers based on Silva database, the bacterial taxonomy of the fecal specimens included 12 phyla and 280 genera. The top dominant bacterial phyla were Bacillota, Bacteroidota (new name of Bacteroidetes), Pseudomonadota, Verrucomicrobia, and Actinomycetota. The dominant bacterial genera were *Bacteroides*, *Blautia*, *Prevotella*, *Akkermansia, Faecalibacterium*, and *Bifidobacterium*.

We found no difference in the average metabolite intensity across the study groups (F = 1.124, df = 2, p = 0.334). In total 23,925 unique metabolomic features were identified in positive ion mode after filtering, consisting of 7077 features with high confidence annotations, and 16,848 unknown features, and 1912 features that mapped to the Kyoto Encyclopedia of Genes and Genomes (KEGG) pathways, the metabolites we focused on this analysis.

### Gut microbiome by study group and antibiotic use

The CWC group at T_0_ (i.e., Shannon [H = 5.88, df = 1, p = 0.015] and Pielou_e [H = 4.22, df = 1, p = 0.04]) and T_1_ (i.e., observed operational taxonomic units [OTUs, H = 4.03, df = 1, p = 0.045], Shannon [H = 4.89, df = 1, p = 0.027], and Pielou_e [H = 3.82, df = 1, p = 0.050]) showed a lower alpha diversity than the HC group. No difference for alpha diversity was found between T_0_ and T_1_ in CWC. CWC receiving antibiotics showed a lower alpha diversity than CWC and HC without receiving antibiotics (i.e., observed OTUs [H = 6.83, df = 1, p = 0.033], Shannon [H = 8.57, df = 1, p = 0.014], and Pielou_e [H = 6.04, df = 1, p = 0.049]). For beta diversity analysis, permutational multivariate analysis of variance (PERMANOVA) based on Jaccard distance and unweighted UniFrac distance showed differences of the CWC at T_0_ (Jaccard [F = 2.0, df = 1, p = 0.003] and unweighted UniFrac [F = 2.06, df = 1, p = 0.027]) and T_1_ (Jaccard [F = 1.93, df = 1, p = 0.001] and unweighted UniFrac [F = 2.15, df = 1, p = 0.015]) from HC groups, as well as between CWC with and CWC and HC without antibiotic use (Jaccard [F = 1.60, df = 1, p = 0.004] and unweighted UniFrac [F = 1.82, df = 1, p = 0.015]). No difference for beta diversity was found between T_0_ and T_1_ in CWC. Principal coordinates analyses visualize beta diversity patterns by study group (Additional file 1: Fig. S1A, B) and by antibiotic use (Additional file 1: Fig. S1C, D).

### Microbiome–metabolome networks linked with PNS

**Fig. 2:**
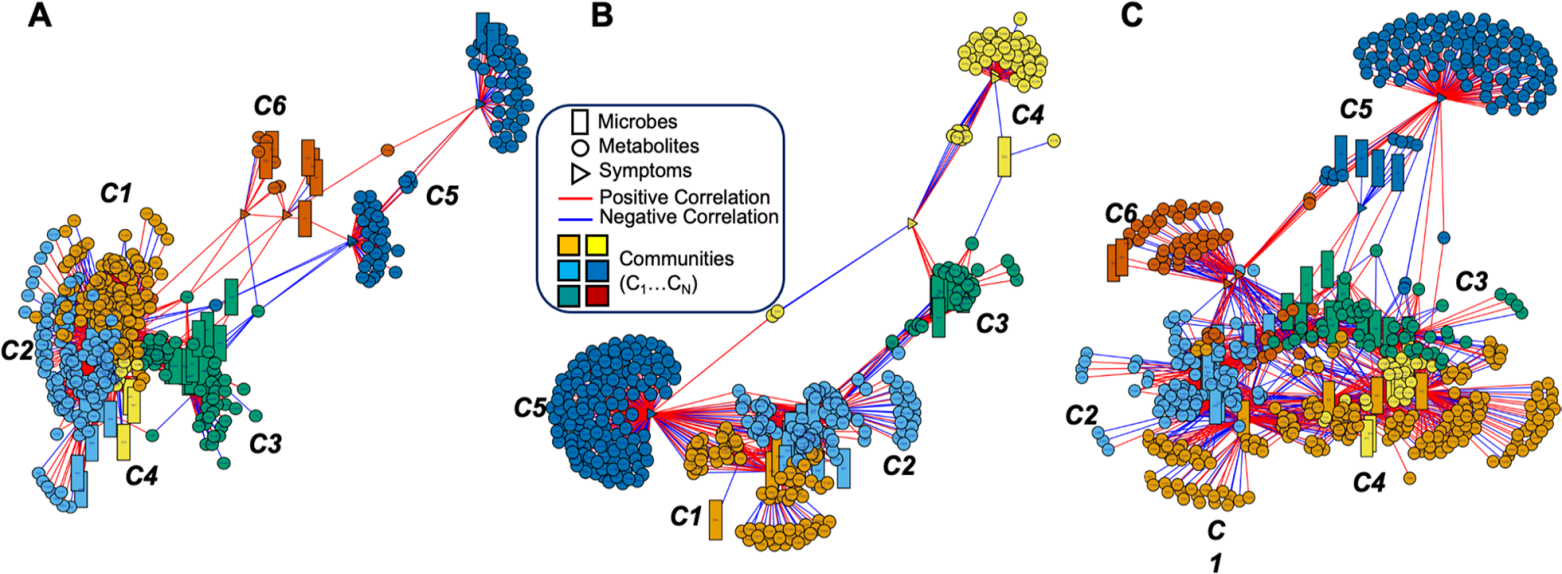
Integrative network analysis of gut microbiome, gut metabolome and psychoneurological symptoms. **A** Presents the network correlations for children pre-cycle two chemotherapy (T_0_), **B** for post-chemotherapy (T_1_), and **C** for healthy controls. The three-way multiomics analysis describes the network relationships among gut microbiome (rectangles), metabolites (circles), and PNS (triangles), including **A** 6 distinct communities (C1…C6) detected for the children with cancer group at T0, **B** 5 distinct communities (C1…C5) detected for the children with cancer group at T1, and **C** 6 distinct communities (C1…C6) detected for healthy controls. Within each panel **A**, **B**, or **C**, Table 2 presents the total number of microbes and metabolites linked with each cluster with specific symptom clusters. Table 4 and Fig. 4 further present these specific gut microbes and metabolites linked with specific clusters with PNS by the study groups

Figure 2 shows the three-way multi-omics network comprised of gut microbiome (rectangles), metabolites (circles), and PNS (triangles) clustered into six distinct communities (C1–C6) for the CWC group at T_0_ (Fig. 2A), five distinct communities (C1–C5) for the CWC group at T_1_ (Fig. 2B), and six distinct communities (C1–C6) for the HC group (Fig. 2C). Further metabolic and microbiome interpretation of Fig. 2 can be found in Tables 2, 3.

**Table 2:** The total number of gut microbiome and metabolites associated with psychoneurological symptoms

Study group	Microbiome	Metabolites
Children with cancer at T_0_ (n = 21)	(n = 37)	(n = 388)
Cluster 1 (no symptoms)	5	121
Cluster 2 (no symptoms)	8	121
Cluster 3 (no symptoms)	12	55
Cluster 4 (no symptoms)	4	12
Cluster 5 (pain, anxiety, cognitive function)	2	71
Cluster 6 (fatigue, depressive symptoms)	6	8
Children with cancer at T_1_ (n = 12)	(n = 24)	(n = 375)
Cluster 1 (no symptoms)	4	71
Cluster 2 (no symptoms)	12	92
Cluster 3 (no symptoms)	7	29
Cluster 4 (fatigue, anxiety, depressive symptoms)	1	48
Cluster 5 (cognitive function)	0	135
Healthy controls (n = 14)	(n = 40)	(n = 492)
Cluster 1 (no symptoms)	4	175
Cluster 2 (no symptoms)	10	75
Cluster 3 (no symptoms)	12	54
Cluster 4 (no symptoms)	7	22
Cluster 5 (fatigue, cognitive function)	5	111
Cluster 6 (anxiety, depressive symptoms)	2	55

**Table 3:** Eigenvector centrality of psychoneurological symptoms in the microbiome–metabolome networks and centrality differences between groups

Symptoms	Children with cancer		Healthy controls (n = 14)	T_1_ vs. T_0_	Healthy controls vs. T_0_	Healthy controls vs. T_1_
	T_0_ (n = 21)	T_1_ (n = 12)				
Pain	0.004	0	0	0.004	0.004	0
Fatigue	0.003	0	0.029	0.003	0.026	0.029
Anxiety	0.003	0	0.167	0.003	0.164	0.167
Depression	0.009	0	0.087	0.009	0.078	0.087
Cognition	0	0.586	0	0.586	0	0.586

T_0_: pre-cycle two chemotherapy; T_1_: post chemotherapy (completion of all chemotherapy)

#### Number of microbial taxa and metabolites linked with PNS

Table 2 shows the total number of significant microbial taxa and metabolites associated with PNS (without any overlap for each cluster) among CWC at T_0_, at T_1_, and HC. Compared to HC, CWC at T_0_ had very similar gut microbial taxa (n [CWC at T_0_] = 37 vs. n [HC] = 40) but a lower number of metabolites (n [CWC at T_0_] = 388 vs. n [HC] = 492). Additionally, CWC at T_1_ showed the lowest number of microbial taxa (n [CWC at T_1_] = 24 vs. n [CWC at T_0_] = 37 vs. n [HC] = 40) and metabolites (n [CWC at T_1_] = 375 vs. n [CWC at T_0_] = 388 vs. n [HC] = 492). Compared with HC and CWC at T_0_, CWC at T_1_ showed a decreased gut microbiome taxa (n [CWC at T_1_] = 1 vs. n [CWC at T_0_] = 8 vs. n [HC] = 7) with increased numbers of metabolites (n [CWC at T_1_] = 183 vs. n [CWC at T_0_] = 79 vs. n [HC] = 166) associated with PNS.

#### Microbiome–metabolome networks linked with PNS

Table 3 shows the eigenvector centrality for PNS in the microbiome–metabolome networks as well as the differences in centrality in the different study groups. Eigenvector centrality scores consider the number of significant correlations with a given feature as well as the number of significant correlations with those correlated features, in turn, with higher scores assigned to features with greater connectivity in the network. The microbiome–metabolome networking nodes were significantly associated with anxiety, depressive symptoms, and fatigue for both CWC at T_0_ and HC. However, the microbiome–metabolome networking nodes were related to pain only for CWC at T_0_. Additionally, the microbiome–metabolome networking nodes were significantly associated with cognition only for CWC at T_1_.

### Specific microbial taxa and metabolic pathways associated with PNS by study group

**Fig. 3:**
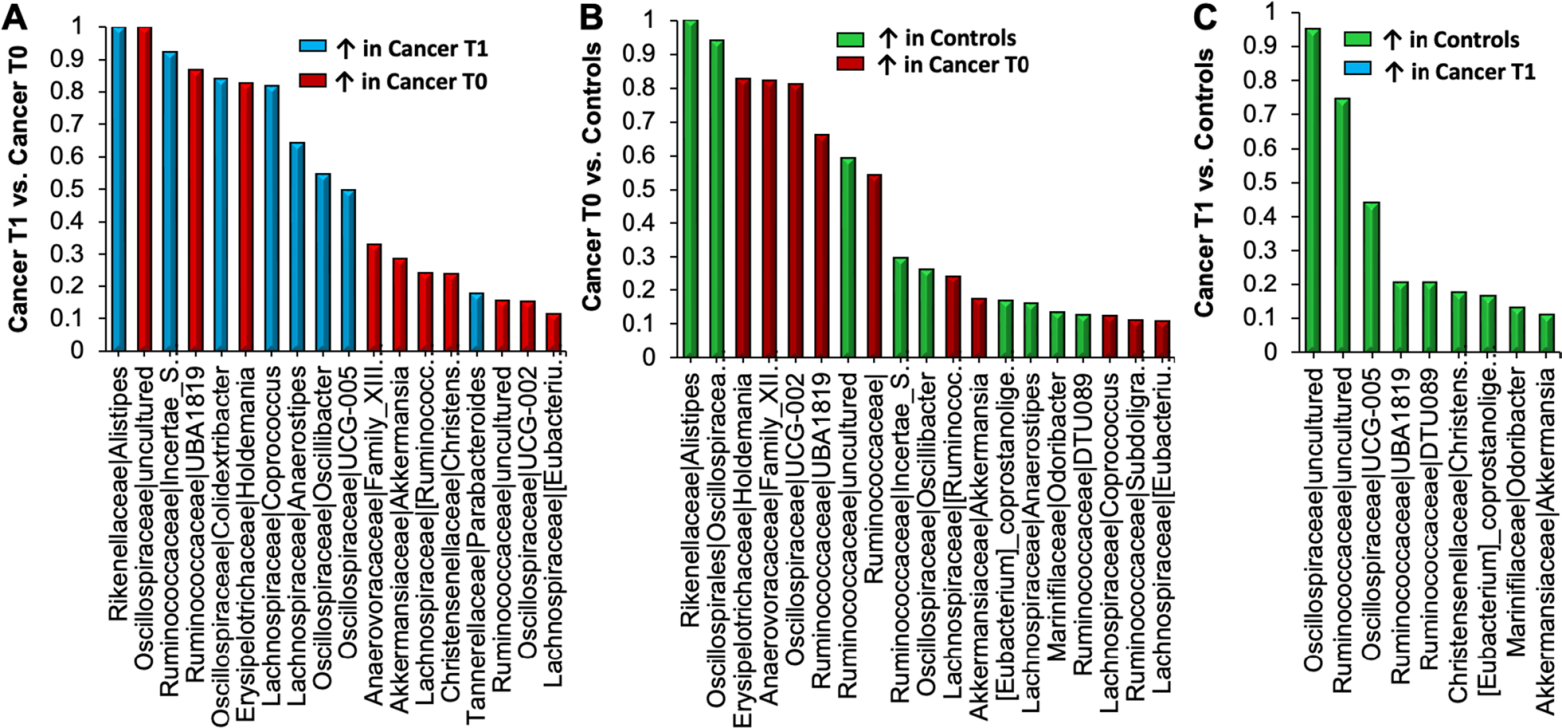
Change in network centrality for the gut microbiome in children with cancer and healthy controls. The gut microbiome with the value of change in network centrality > 0.1 were selected via comparisons among cancer pre-cycle two chemotherapy (T_0_), cancer post-chemotherapy (T_1_) and healthy controls. **A** Presents T_0_ vs. T_1_; **B** presents T_0_ vs. controls; and **C** presents T_1_ vs. controls. No microbes were found significantly higher for cancer T_1_

Figure 3 demonstrates changes in network centrality for the gut microbiome in our samples by selecting the value of change in network centrality > 0.1. Changes in network centrality can indicate altered interactions among the features in different states. The genera in CWC that were more abundant at T_0_ than T_1_ included *UBA1819, Holdemania*, *Anaerovoracaceae_Family_XIII*, and *Akkermansia*. Increased abundance of *Alistipes*, *Incertae_S*, *Colidextribacter*, *Coprococcus*, and *Anaerostipes* genera were found in CWC at T_1_ relative to T_0_ (Fig. 3A). Compared to CWC at T_0_, HC had increased abundance of *Alistipes*, *Oscillospiracea*, *Incertae_S*, and *Oscilllibacter* genera (Fig. 3B). Furthermore, compared to CWC at T_1_, HC had increased abundance of *UCG-005*, *UBA1819*, *DTU089*, *Christensenellaceae R7 group*, *Eubacterium_coprostanoligenes group*, *Odoribacter*, and *Akkermansia* genera (Fig. 3C).

#### Children with cancer at T_0_

**Fig. 4:**
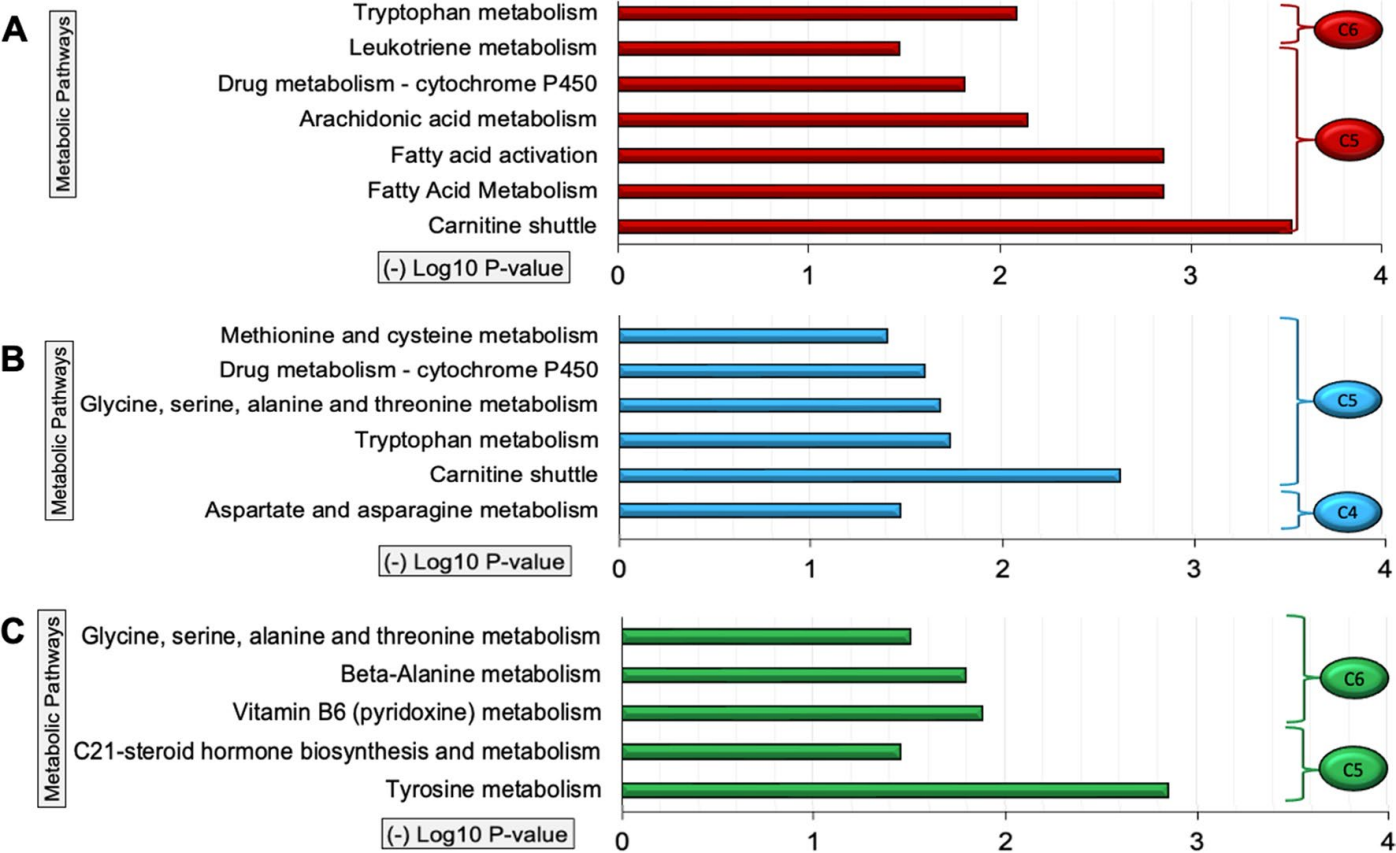
Integrative network analysis of fecal metabolome associated with PNS. **A** Presents CWC at T_0_; **B** presents CWC at T_1_; and **C** presents healthy controls

PNS were assigned to two microbe–metabolite–PNS communities, C5 (pain, anxiety, and cognitive dysfunction) and C6 (fatigue and depressive symptoms) (Fig. 2A and Table 2). Within those communities, PNS were negatively associated with gut microbes (e.g., *Lactobacillus*, *Bifidobacterium,* and *Roseburia*), which are identified with probiotic functions or SCFA producers. These gut microbes were also associated with metabolic pathways of carnitine shuttle (p = 0.0003), fatty acid metabolism (p = 0.001) and activation (p = 0.001), and tryptophan metabolism (p = 0.008) (Table 4 and Fig. 4A).

**Table 4:** Gut microbiome associated with the node of metabolome-psychoneurological symptom cluster

Study group	Symptom cluster	Gut microbial genera
Children with cancer at T_0_	Cluster 5	*Ruminococcus*, *Parasutterella*
	Cluster 6	*Parabacteroides*, *Veillonella*, *Megasphaera*, *UBA1819*, *Escherichia–Shigella*, *Prevotella*
Children with cancer at T_1_	Cluster 4	*Megasphaera*
Healthy controls	Cluster 5	*Lachnoclostridium*, *UBA1819*, *Coprococcus*, *Prevotella*, *Alistipes*
	Cluster 6	*Anaerostipes*, *Phascolarctobacterium*

T_0_: pre-cycle two chemotherapy; T_1_: post chemotherapy (completion of all chemotherapy)

#### Children with cancer at T_1_

PNS were assigned to two microbe–metabolite–PNS communities, C4 (fatigue, anxiety, and depressive symptoms) and C5 (cognitive function) (Fig. 2B and Table 2). Within those communities, PNS were negatively associated with *Intestinibacter* and *Megasphaera* genera, which were also associated with aspartate and asparagine metabolism (df = 1, p = 0.034), carnitine shuttle (df = 1, p = 0.002), tryptophan (df = 1, p = 0.019), glycine, serine, alanine, and threonine metabolism (df = 1, p = 0.020), drug metabolism-cytochrome P450 (df = 1, p = 0.025), and methionine and cysteine metabolism (df = 1, p = 0.040) (Table 4 and Fig. 4B).

#### Healthy controls

PNS were assigned to two microbe–metabolite–PNS communities, C5 (fatigue and cognitive function) and C6 (anxiety and depressive symptoms) (Fig. 2C and Table 2). Within those communities, PNS were negatively associated with gut microbes (e.g., *Ruminococcaceae_UBA1819*, *Coprococcus, Prevotella, Alistipes,* and *Lachnoclostridium*) which were also significantly associated with metabolic pathways of tyrosine metabolism (df = 1, p = 0.001), C21-steroid hormone biosynthesis and metabolism (df = 1, p = 0.035), vitamin B6 (pyridoxine) metabolism (df = 1, p = 0.013), β-alanine metabolism (df = 1, p = 0.016), and glycine, serine, alanine, and threonine metabolism (df = 1, p = 0.032) (Table 4 and Fig. 4C).

#### Comparison between children with cancer and controls

A comparison of microbiome–metabolome–PNS networks between CWC and HC groups elucidated different impact patterns of bacteria with potential probiotic functions (e.g., *Ruminococcaceae* and *Akkermansia*) and fatty acid metabolism, tryptophan, and carnitine shuttle on the microbiome–metabolome–PNS networks. Multiple comparison corrections were not implemented in this study considering its exploratory nature, which is a limitation of this study.

## Discussion

This study examined the microbiome–metabolome pathways associated with PNS among CWC receiving chemotherapy compared to HC in a network-based multi-omics analysis. We found that CWC post-chemotherapy showed the lowest number of correlated gut microbes, but more metabolites compared with those pre-cycle two chemotherapy and HC. Different patterns of microbiome–metabolite–PNS networks post-chemotherapy are associated with changes of PNS trajectories and the disturbed gut microbiome across cancer chemotherapy. Interestingly, PNS were clustered into two communities within the microbiome–metabolome networks in both study groups, revealing that specific gut microbial genera (e.g., *Megasphaera*, *Ruminococcus*, and *Prevotella*) were associated with the carnitine shuttle, fatty acid metabolism/activation, and tryptophan metabolic pathways. As the first of its kind, this study identified microbiome–metabolome pathways associated with PNS for CWC using a multi-omics approach. Although this study was limited by a small sample size, our findings provide promising microbiome–metabolome targets to validate in future studies with larger cohorts.

Compared with HC, CWC receiving chemotherapy reported more symptom burden, with particularly increased fatigue and cognitive dysfunction scores. Our findings partially reflected previous work regarding the overall trend of PNS across chemotherapy [51, 52], including potential improvement of some symptoms, such as pain [53, 54] and anxiety [54] post-chemotherapy. This may be attributed to recovery from treatment-related procedures, acute chemotherapy toxicities, and discharge from the hospital after completion of chemotherapy. However, the worsened fatigue and cognitive dysfunction post-chemotherapy echo previous literature on the continuity of chemotherapy adverse events, particularly severe fatigue [55] and cognitive impairment [56, 57] in this population. Due to the significant influence of PNS on children’s future functional status and QOL, understanding the biological mechanisms of PNS trajectories during chemotherapy treatment is critical.

Recent innovations in the MGB axis propose that the gut microbiome can influence PNS via regulating specific metabolic pathways (e.g., SCFAs and tryptophan) [12, 18, 58]. Chemotherapy has been reported to disturb the gut microbiome in CWC, reducing abundance of anaerobic bacteria (i.e., *Bacteroides*, *Clostridium cluster XIVa*, *Faecalibacterium*, and *Bifidobacterium*), whereas *Enterococcus*, often pathogenic, drastically increased [31, 59, 60]. The disturbed gut microbiome potentially heightens treatment-related toxicity [61]. Although limited, specific gut microbial taxa were found associated with PNS among adult cancer patients [13, 16, 24]. Patients with a high PNS cluster burden were more likely to have increased abundance of Bacteroidota and *DTU089* phyla and *Ruminiclostridium-9*, *Tyzzerella*, *Eubacterium_fissicaten* genera, while those with a low PNS cluster burden had higher abundance of *Lactococcus*, *Phascolarctobacterium*, and *Desulfovibrio* genera*.* Our study found an increase in the *Akkermansia* genus for CWC pre-cycle two chemotherapy and HC, which was negatively linked to PNS in the microbiome–metabolome networks. Findings of this study support that higher abundance of *Akkermansia* is associated with lower PNS burden, particularly cognitive dysfunction among CWC post-chemotherapy. Similarly, decreased abundance of *Akkermansia* species is associated with various adverse health effects, including metabolic disorders, inflammatory and neurodegenerative diseases, and even cancers [62, 63]. Having a protective effect, *Akkermansia* species can act on host metabolism and metabolites such as SCFAs [64]. For example, the probiotic *Akkermansia muciniphila* is well known as a propionate producer in the presence of vitamin B12 [65]. Although the mechanism of *Akkermansia* species on disease and health outcomes is largely unknown, current key hypothesis is the positive modulation of thickness of intestinal mucosa and intestinal barrier integrity [66, 67]. For instance, patients with cancer experienced significant epithelial permeability and bacterial translocation [68]. Thus, therapeutic manipulations (e.g., probiotics and Mediterranean diet) of the *Akkermansia* species may maintain the intestinal integrity [67] and further reduce chemotherapy-related PNS.

In this study, gut microbial genera, such as *Lactobacillus*, *Bifidobacterium*, and *Roseburia* taxa were associated with lower PNS burden [69, 70]. Decreased abundances of the gut microbial taxa, particularly *Roseburia* and *Faecalibacterium* were commonly reported among patients with psychiatric disorders [71]. Although mixed findings using non-experimental study designs (e.g., case–control and observational) were reported about *Bifidobacterium* and *Lactobacillus* among patients with psychiatric disorders [71–73], probiotic interventions seem to support *Bifidobacterium* and *Lactobacillus* species (e.g., *Lactobacillus rhamnosus* and *Bifidobacterium breve*) as an alternative therapy to alleviate PNS (e.g., anxiety, depression, and cognitive dysfunction [74–77]). Due to methodological shortcomings, further confirmation of these findings is critically needed. Additionally, the gut microbes (e.g., *Ruminococcaceae_UCG-014*) [78] are associated with lower PNS burden. However, there has also been contradicting evidence regarding probiotics, such as *Alistipes*. They may have protective effects in the PNS context but have also been demonstrated to have a pathogenic nature associated with the development of colorectal cancer and depression [79]. Thus, our findings require further confirmation in a larger cohort of pediatric cancer patients.

Gut microbes metabolize dietary and host-derived molecules to activate or produce functional metabolites with local and systemic effects [26]. Under the guidance of the MGB axis framework, our previous research identified a group of serum metabolites associated with pain, fatigue, anxiety, depressive symptoms, and the PNS cluster (mean of these symptoms) for CWC (e.g., primarily diagnosed with leukemia and lymphoma) across a chemotherapy cycle [14]. In particular, the fatty acid pathways were associated with pain, the tryptophan pathway was associated with fatigue, anxiety, and the PNS cluster, and the carnitine shuttle was associated with the PNS cluster [14]. Furthermore, a dysbiotic gut microbiome was found to potentially modulate PNS through altered lipid metabolism as well as gastrointestinal and neural systems for patients with head and neck cancer [13]. This study compared microbiome–metabolome–PNS networks among CWC pre-cycle two chemotherapy and post-chemotherapy, and HC and indicated that different patterns of bacteria (e.g., *Ruminococcus* and *Prevotella*) linked with metabolites (e.g., fatty acid metabolism, tryptophan, and carnitine shuttle) are associated with PNS by study groups. These network differences may be partially attributed to the effects of chemotherapy and antibiotic use, which can shape the gut microbiome, and in turn further aggravate dysregulations of metabolic pathways, intestinal permeability, and damage to the enteric and peripheral nerves, ultimately leading to physiological and psychological dysfunction [13, 80, 81]. Specifically, *Ruminococcus* has been reported to form secondary bile acid (e.g., ursodeoxycholic acid) that modulates the immune system via reducing cytokine secretion and inhibiting eosinophil activation [82]; *Prevotella* has also been reported to produce SCFAs and add in the synthesis of micronutrients (e.g., vitamin K2 and B12) [83, 84], which can regulate intestinal homeostasis in animal models and human populations. The findings of microbiome–metabolome–PNS networks provide potential targets (e.g., microbes and its functional metabolites) to mitigate PNS for children with cancer receiving chemotherapy. For example, administrating probiotics (e.g., *Lactobacillus* and *Bifidobacterium*) can correct microbial dysbiosis and sustain metabolic equilibrium [85]. Although more work is needed to confirm our findings in CWC, our findings from this study suggest that microbiome–metabolome pathways are associated with PNS among children with cancer receiving chemotherapy.

Single-omics biomarkers (i.e., microbiome or metabolome) are emerging to explain PNS in cancer chemotherapy [14, 24, 25]. However, there is a paucity of research that integrates both the gut microbiome and metabolome using multi-omics approaches in PNS. Consistent with our previous work [14], this study found that carnitine shuttle, fatty acid activation and metabolism, and tryptophan metabolism were associated with the gut microbiome and PNS in CWC. Specifically, carnitine is a trimethylated amino acid primarily derived from the diet, essential for the transportation of long-chain fatty acids during fatty acid beta-oxidation for energy support, including cancer [86–88]. An interruption of the carnitine shuttle system during chemotherapy could influence cancer metabolic plasticity and intertwine key metabolic pathways that supply an energetic and biosynthetic demand for cancer cells [89], ultimately influencing PNS during chemotherapy. Considering the critical role of carnitine-related pathways in cancer care, l-carnitine supplementation was explored to improve PNS, particularly fatigue [90, 91].

Additionally, we found that fatty acid activation and metabolism involved in the carnitine shuttle system were associated with PNS. Fatty acids metabolism includes various metabolic processes involving fatty acids, which determine human brain’s integrity and functional performance [92]. Essential fatty acids, such as omega-3 fatty acids, were found to decrease the symptoms of fatigue and pain in patients during chemotherapy, possibly due to weight maintenance and reduced inflammatory status [93]. Furthermore, a decrease in bile acid synthesis was reported in patients with chronic fatigue syndrome [94]. This may be attributed to the role of bile acids in cholesterol homeostasis and microbiome signaling, facilitating excretion, absorption, and transportation of fat and sterols in the liver and intestines [95]. Together, specific fatty acids, such as omega-3 fatty acids, point towards a precision approach to treat and manage cancer treatment-related symptoms although further investigation is needed to examine the exact benefits of fatty acid-related supplementations or diets rich in omega-3 and omega-6 fatty acids in symptoms among cancer populations, including children with cancer [93, 96].

Tryptophan, an essential amino acid, is required for structural and functional processes of protein biosynthesis and immunoregulation [97] and plays a critical role in the MGB axis [98]. The inflammation activation of tumor cells and cancer treatments can induce the tryptophan-degrading enzyme indoleamine 2,3-dioxygenase, which can convert tryptophan to kynurenine in the gastrointestinal tract and other tissues of the body [99]. Downstream metabolites of kynurenine include neuroprotective kynurenic acid and neurotoxic quinolinic acid [18]. Depletion of tryptophan could contribute to serotonin dysregulation and neurobehavioral manifestations [100, 101]. Meanwhile, the accumulation of downstream metabolites of the kynurenine pathway seems to trigger central nervous system physiology, anxiety, depression, social behavior, cognition, and visceral pain [18, 102]. Similarly, they were also associated with an increased burden of pain, fatigue, anxiety, and depression [14, 103, 104], as well as reduced QOL [105, 106]. This study corroborated previous reports that demonstrate the association of altered tryptophan metabolism during chemotherapy and its adverse association with symptom burden among CWC. Current literature has attempted to identify solutions to inhibit tryptophan breakdown, such as ketogenic diet [107], Mediterranean and other plant-based diets [108], probiotics [109], and physical activity [110, 111]. Further studies are needed to test the feasibility and efficacy of these promising interventions among pediatric cancer populations.

Utilizing the MGB axis framework, this study confirmed several metabolic pathways, such as carnitine shuttle and tryptophan/kynurenine pathways, associated with psychoneurological toxicities in children [14, 112] and adults with cancer undergoing chemotherapy [24, 113, 114]. This is the first study to elucidate microbiome–metabolome pathways linked with PNS in cancer chemotherapy using the multi-omics data integration and analysis approach. This study added to the literature that specific gut microbes (e.g., *Ruminococcus*, *Megasphaera*, and *Prevotella*), along with carnitine shuttle, fatty acid metabolism/activation, and tryptophan pathways, are associated with PNS burden across cancer chemotherapy. Targeting the gut microbiome through diet, nutritional supplements, probiotics, and exercise [18, 115] may provide a tractable solution to modulate metabolic pathways, ultimately decreasing PNS burden among CWC. Further validation of these findings is needed in a larger cohort.

There are several limitations to our study. First, the sample size is small, and all cases were recruited from Children’s Healthcare of Atlanta, resulting in limited generalizability into other clinical settings. This pilot study analyzed CWC who completed T_0_ and some of them did not complete T_1_ yet when we analyzed the data. The unbalanced sample size between T_0_ and T_1_ for CWC may cause bias. Second, as a preliminary analysis with a smaller sample size, we did not adjust the multi-omics integration for multiple testing. This approach has certainly resulted in some false positive findings, furthering the importance of future replication. However, clustering and pathway analyses are two ways to mitigate the effects false positives in omics research [116], and our prior research suggests that these approaches might continue to do so in multi-omics research [117]. Future work should confirm our findings in a larger cohort with multiple testing correction. Third, we were unable to determine whether the fecal metabolites were produced by the microbiome or by the host, and whether these identified metabolites were being absorbed to affect the MGB axis or alternatively being eliminated. Our metabolomics analysis was limited to summaries of metabolic pathways and thus need detailed examination of specific metabolites in future work to determine the magnitude and direction. Lastly, our study could not control for the use of antibiotics and chemotherapy drugs across the cancer treatment trajectory. Therefore, this study cannot determine the impact of specific chemotherapy on PNS and the antibiotic vs. chemotherapy effects on microbiome–metabolome pathways. We cannot discern baseline differences in the fecal microbiome and metabolome due to treatments or cancer per se. Future research should examine the relationships of multi-omics pathways in the chemotherapy-induced PNS context with a larger sample cohort using metagenomic sequencing to elucidate species- and strain-level microbial data, as well as targeted metabolomics that focus on the most salient pathways (e.g., tryptophan), while controlling for covariates such as chemotherapy drugs.

## Conclusion

CWC seemed to report more symptom burden than HC, particularly with more fatigue and cognitive dysfunction post-chemotherapy. With the support of the MGB axis, our multi-omics analyses identified specific gut microbial genera clustered with carnitine shuttle, fatty acid metabolism/activation, and tryptophan pathways are associated with PNS burden across cancer chemotherapy. The trend of symptom burden and its association with microbiome–metabolome pathways should be further validated in a large cohort. These findings can guide clinical practices via informing the development of novel interventions targeting microbiome–metabolome pathways (e.g., prebiotics, probiotics, and physical activity) [17, 118] to relieve symptom burden in children with cancer.

### Supplementary Information

**Additional file 1: Figure S1.** Beta Diversity of the Gut Microbiome by Group and Antibiotic Use. A and B present the microbial dissimilarity by study group and antibiotic use based on Jaccard distance. C and D present the dissimilarity by study group and antibiotic use based on unweighted UniFrac distance. HC, healthy control; CWC, children with cancer. T_0_, pre-cycle two chemotherapy; T_1_, post-chemotherapy.

## Data Availability

Part of the data have been released in NCBI database with dbGaP Study Accession: phs002960.v1.p1. The complete datasets used during the current study are available from the corresponding author on reasonable request.
