# Supplementary material for: Multi-omics analysis of the gut microbiome and metabolites associated with the psychoneurological symptom cluster in children with cancer receiving chemotherapy
Source: J Transl Med. 2024 Mar 9;22:256. doi: 10.1186/s12967-024-05066-1 (PMC10924342; doi:10.1186/s12967-024-05066-1)
Supplement: Supplementary file 1 — Additional file 1: Figure S1. Beta Diversity of the Gut Microbiome by Group and Antibiotic Use. A and B present the microbial dissimilarity by study group and antibiotic use based on Jaccard distance. C and D present the dissimilarity by study group and antibiotic use based on unweighted UniFrac distance. HC, healthy control; CWC, children with cancer. T0, pre-cycle two chemotherapy; T1, post-chemotherapy. [file 12967_2024_5066_MOESM1_ESM.docx]

**Additional file 1**

**Additional Figure**

**Figure S1.** Beta-Diversity of the Gut Microbiome by Study Group and Antibiotic Use. A and B present the gut microbial dissimilarities by study group and antibiotic use using Jaccard distance. C and D present the dissimilarities by study group and antibiotic use using unweighted UniFrac distance. HC, healthy control; CWC, children with cancer. T_0_, pre-cycle two chemotherapy; T_1_, post-chemotherapy.

**
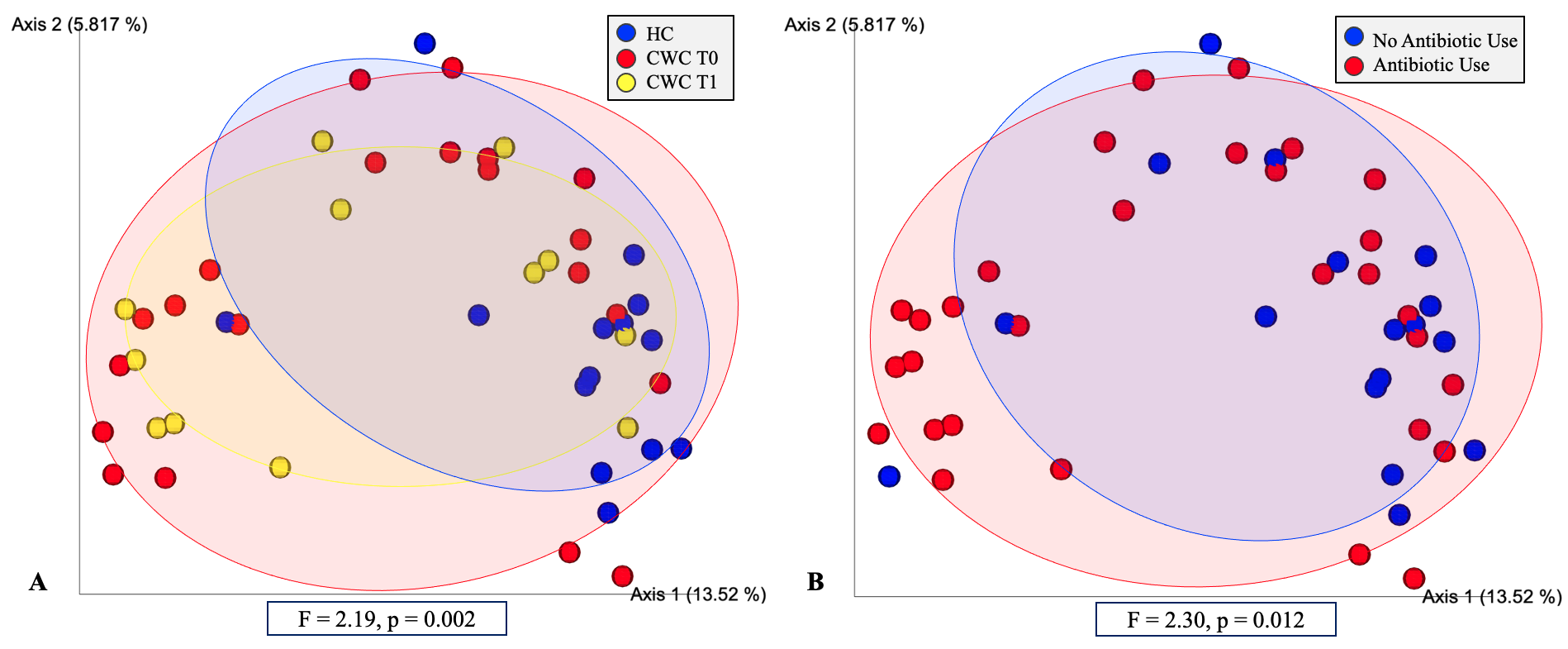
**

**
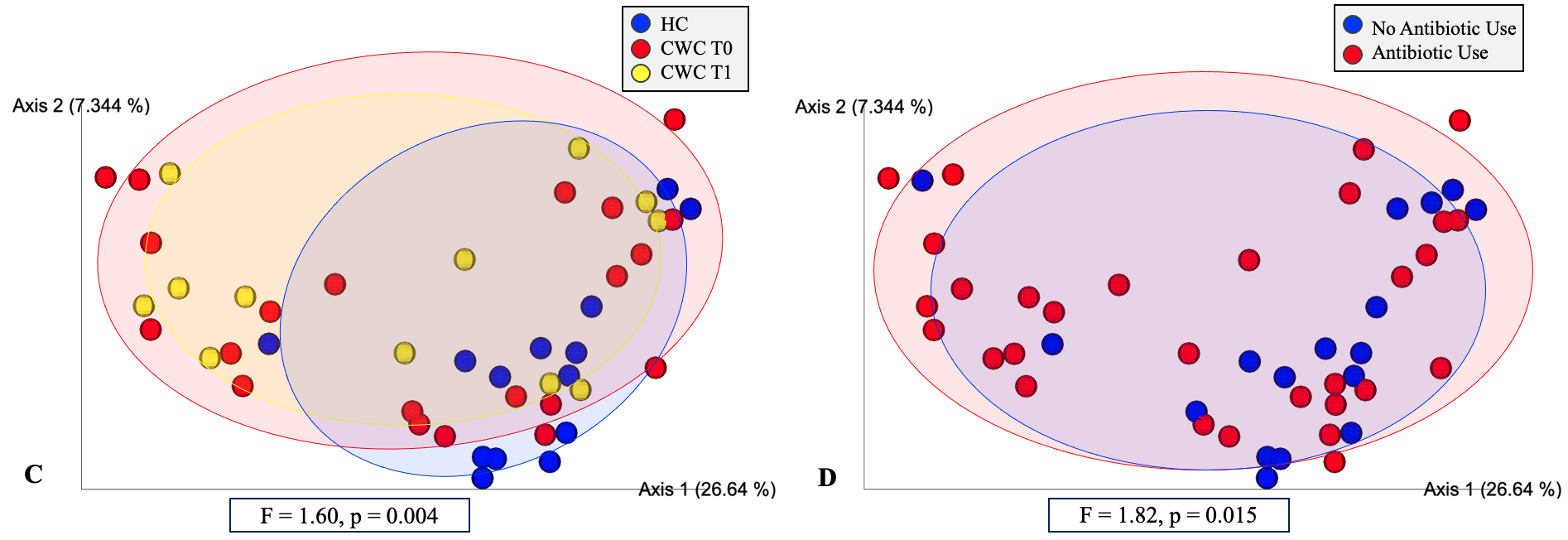
**
